# Supplementary material for: Transcriptomic Insights into Mechanisms of Early Seed Maturation in the Garden Pea (Pisum sativum L.)
Source: Cells. 2020 Mar 23;9(3):779. doi: 10.3390/cells9030779 (PMC7140803; doi:10.3390/cells9030779)

Sprint−2,10(4)

Global Summary

%DE = 0.27

genes with fdr < 0.2 = 169 (88 + / 81 −)

genes with fdr < 0.1 = 158 (83 + / 75 −)

genes with fdr < 0.05 = 153 (80 + / 73 −)

genes with fdr < 0.01 = 135 (72 + / 63 −)

genes in genesets = 0

<FC> = 0

<t−score> = 0

<p−value> = 0

<fdr> = 0.73

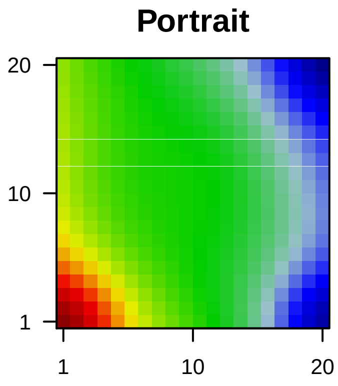

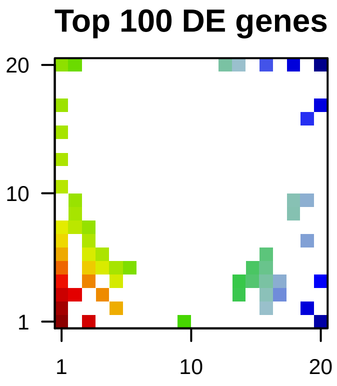

Global Genelist

Rank	ID	log(FC)	p−value	fdr	Metagene	Description
1	TRINITY_DN1	0.23	2e−16	1e−15	1 x 2	
2	TRINITY_DN3	0.36	2e−16	1e−15	3 x 5	
3	TRINITY_DN1	−13.29	2e−16	1e−15	20 x 1	
4	TRINITY_DN1	−8.1	2e−16	1e−15	20 x 20	
5	TRINITY_DN3	0.22	2e−16	1e−15	1 x 17	
6	TRINITY_DN2	−3.14	2e−16	1e−15	20 x 1	
7	TRINITY_DN2	−1.59	2e−16	1e−15	20 x 1	
8	TRINITY_DN3	1.81	2e−16	1e−15	1 x 1	
9	TRINITY_DN3	−0.68	2e−16	1e−15	20 x 17	
10	TRINITY_DN3	−0.25	2e−16	1e−15	19 x 10	
11	TRINITY_DN5	1.42	2e−16	1e−15	1 x 1	
12	TRINITY_DN4	4.64	2e−16	1e−15	1 x 1	
13	TRINITY_DN4	−0.47	2e−16	1e−15	18 x 20	
14	TRINITY_DN1	0.55	2e−16	1e−15	1 x 1	
15	TRINITY_DN3	−1.31	2e−16	1e−15	20 x 1	
16	TRINITY_DN2	0.65	2e−16	1e−15	1 x 2	
17	TRINITY_DN2	−0.94	2e−16	1e−15	20 x 20	
18	TRINITY_DN2	0.93	2e−16	1e−15	1 x 1	
19	TRINITY_DN4	2.33	2e−16	1e−15	1 x 1	
20	TRINITY_DN1	−0.22	2e−16	1e−15	16 x 5	

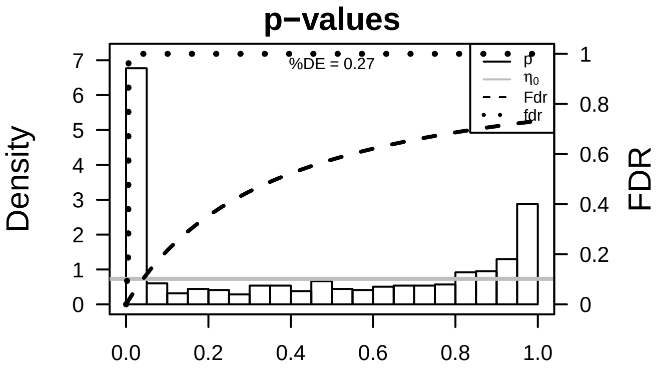

Supplement: Supplementary file 1 [file cells-09-00779-s001.zip › Supplementary materials/FileS3.zip!/FileS2/Hormones - Results/Summary Sheets - Samples/Sprint.2.10.4..pdf]
